# Supplementary material for: Multi-omics regulatory network inference in the presence of missing data
Source: Brief Bioinform. 2023 Sep 5;24(5):bbad309. doi: 10.1093/bib/bbad309 (PMC10516394; doi:10.1093/bib/bbad309)
Supplement: 230410_suppl_figs_bbad309 [file 230410_suppl_figs_bbad309.pdf]

Supplementary Figures

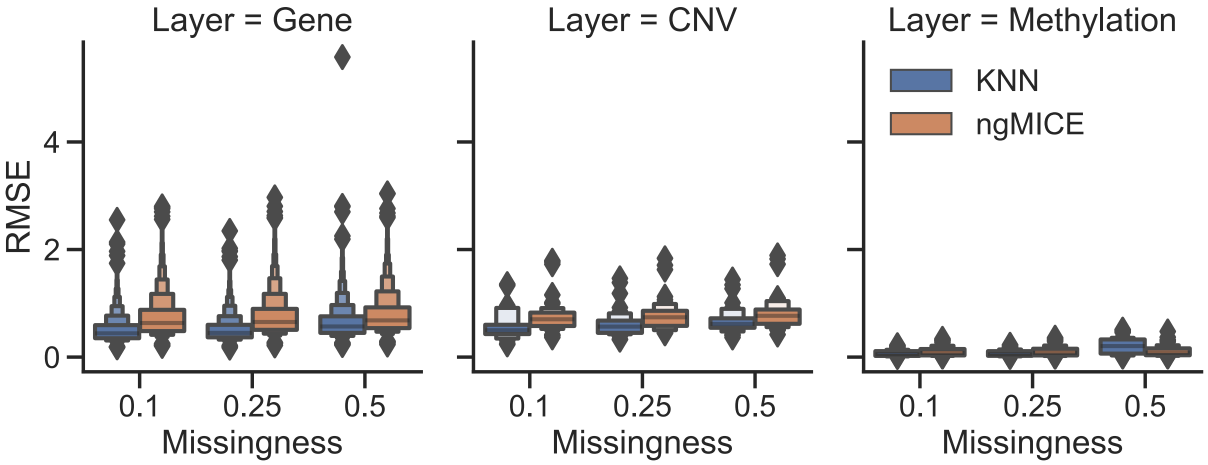

Supplementary Figure 1. Root means square mean error of kNN-based and network-based multiple imputation per omics layer with increased missingness (10%, 25%, 50%) removing randomly elements from each omics individually.

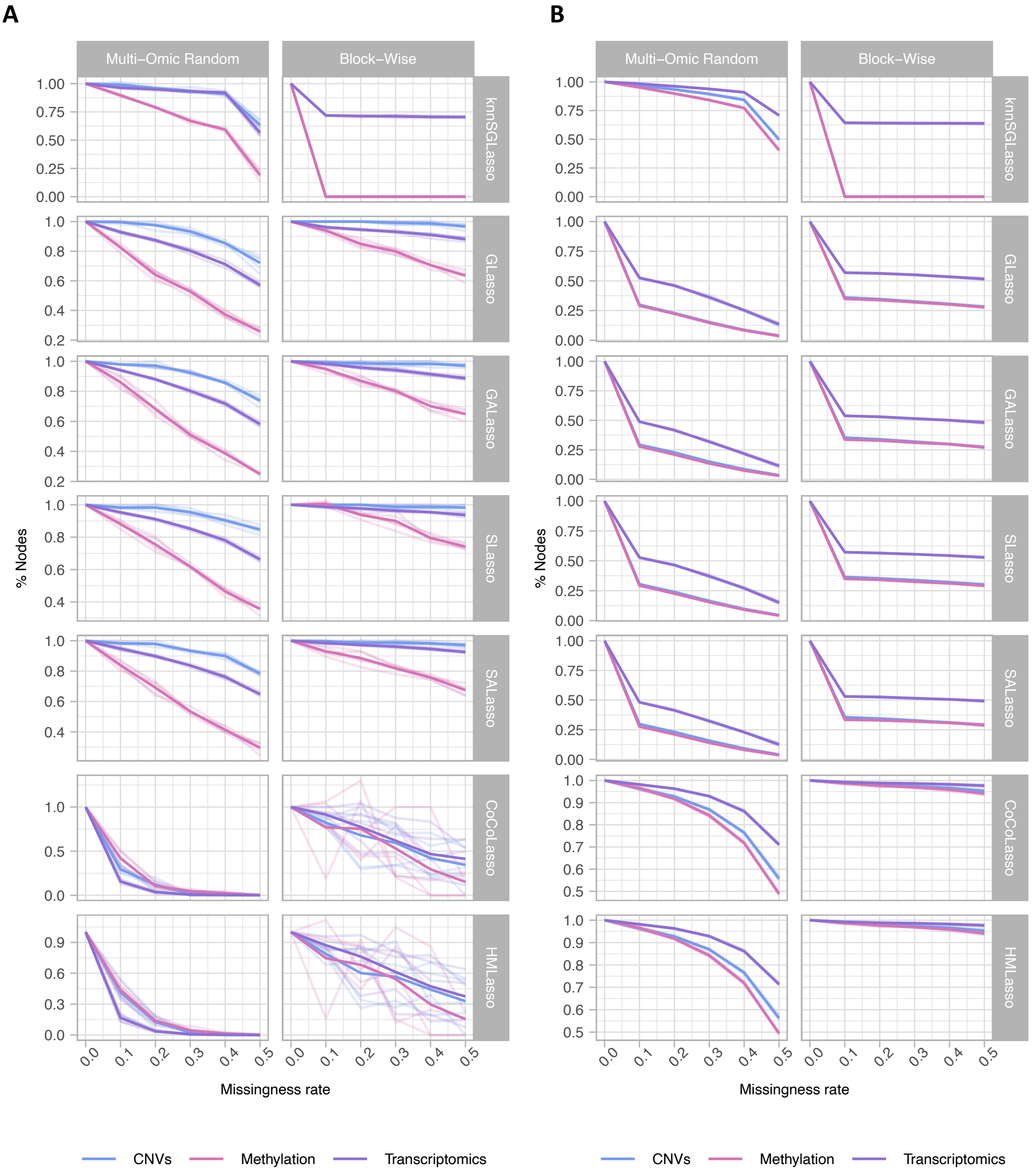

Supplementary Figure 2. Feature reduction per omics layer represented as the percentage loss compared to the full-data network inference, **A)** TCGA-BRCA, **B)** TCGA-MIBC.

A

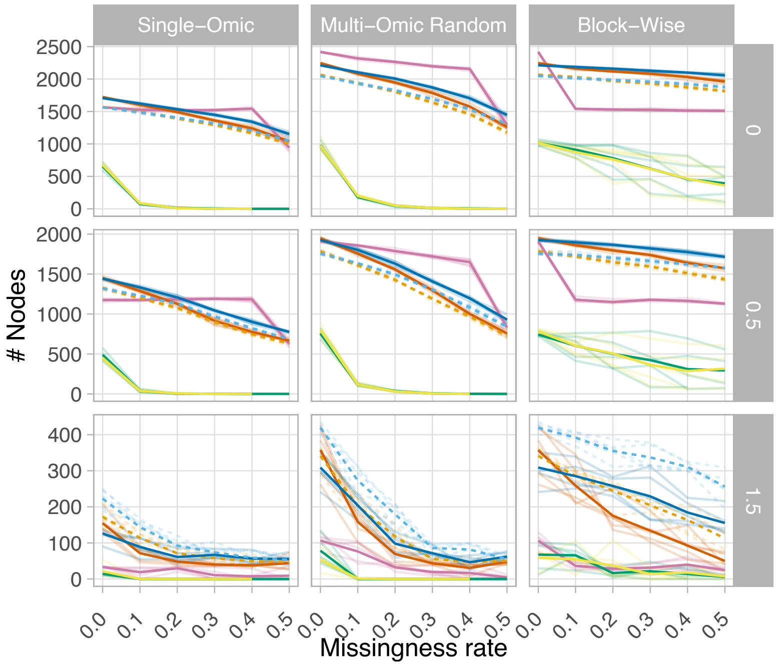

B

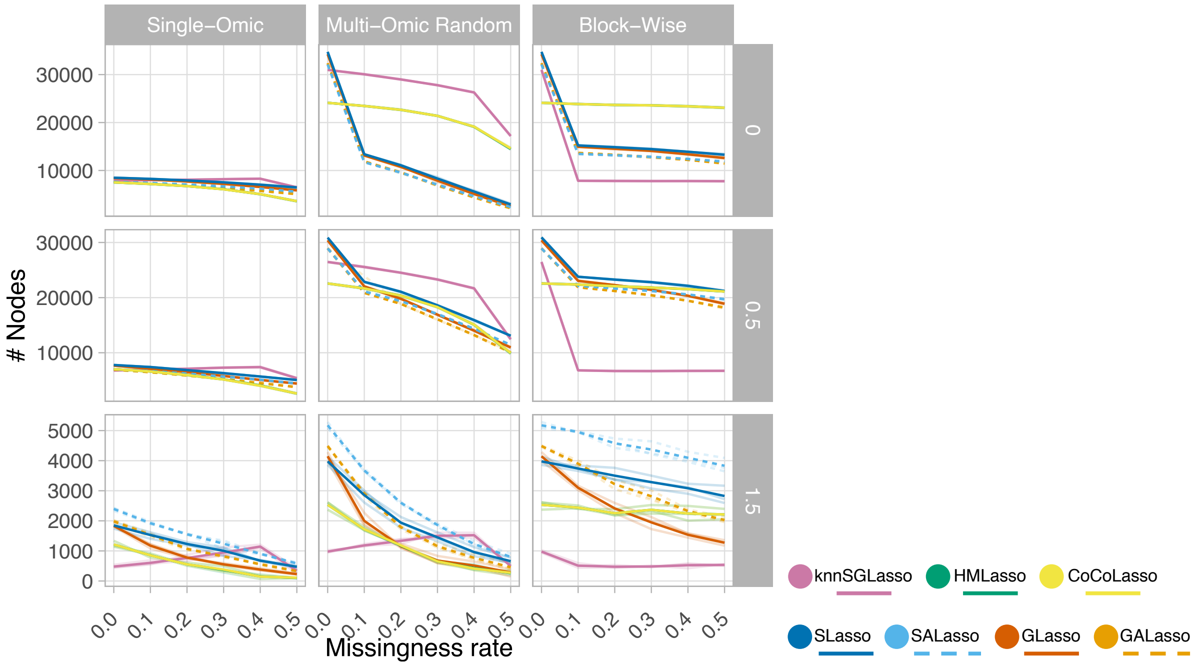

Supplementary Figure 3. Number of nodes indicating network size across all tested noise levels, **A)** TCGA-BRCA, **B)** TCGA-MIBC.

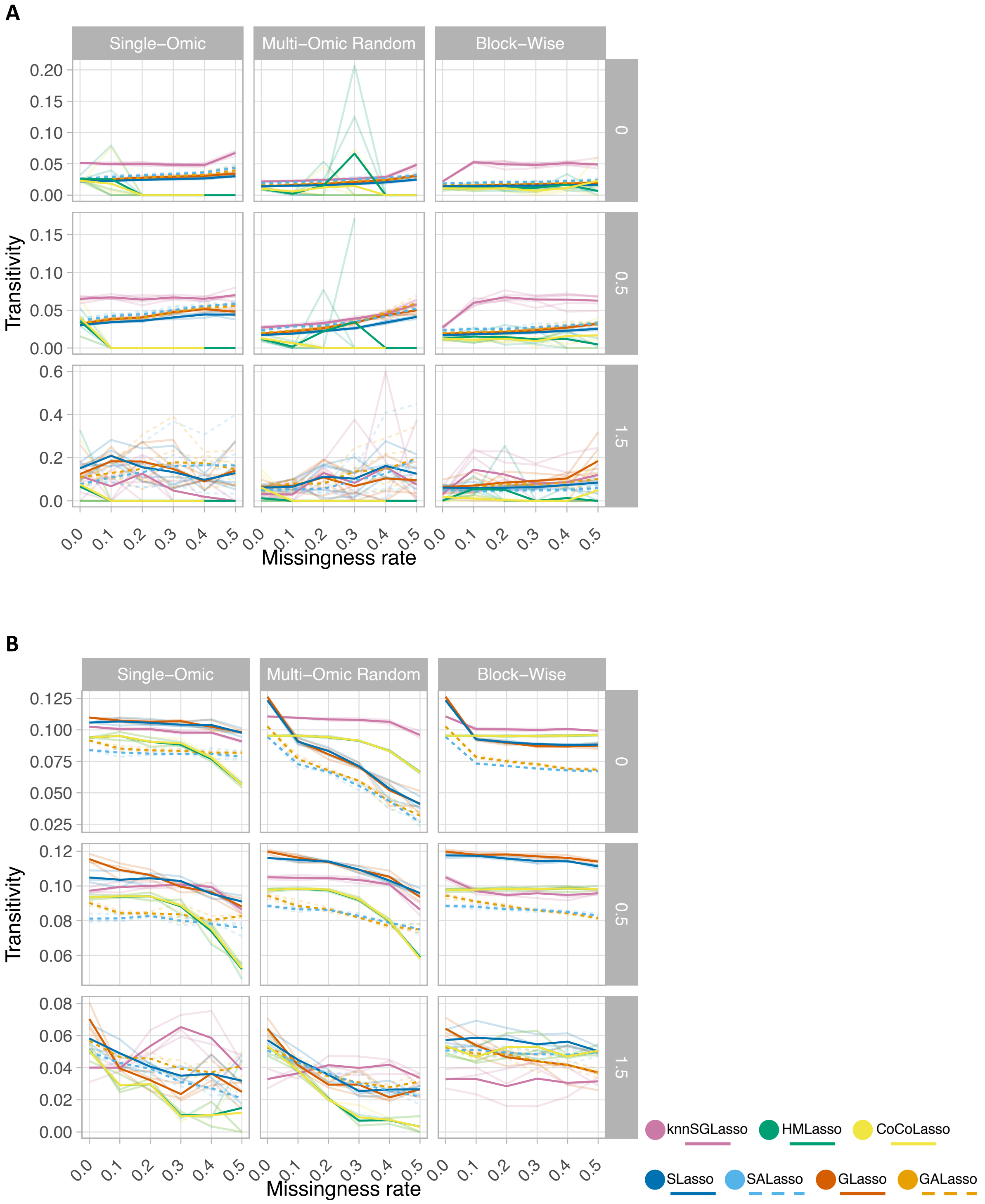

Supplementary Figure 4. Global clustering coefficient per network inferred. **A)** TCGA-BRCA, **B)** TCGA-MIBC.

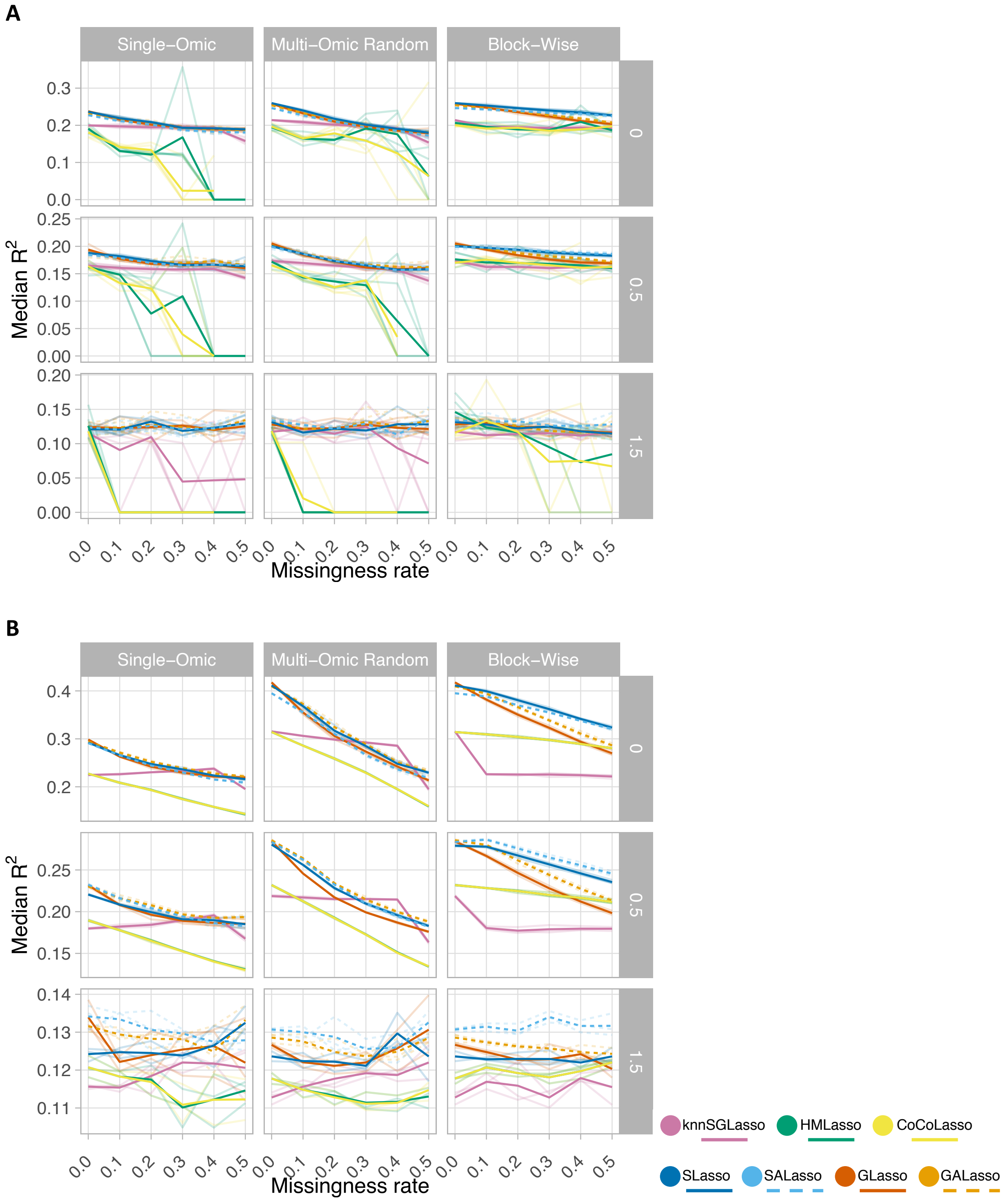

Supplementary Figure 5. Performance evaluation via R^2. For each network all nodes are evaluated via R^2. A single networks' quality can be estimated via median calculation across all models, **A)** TCGA-BRCA, **B)** TCGA-MIBC.

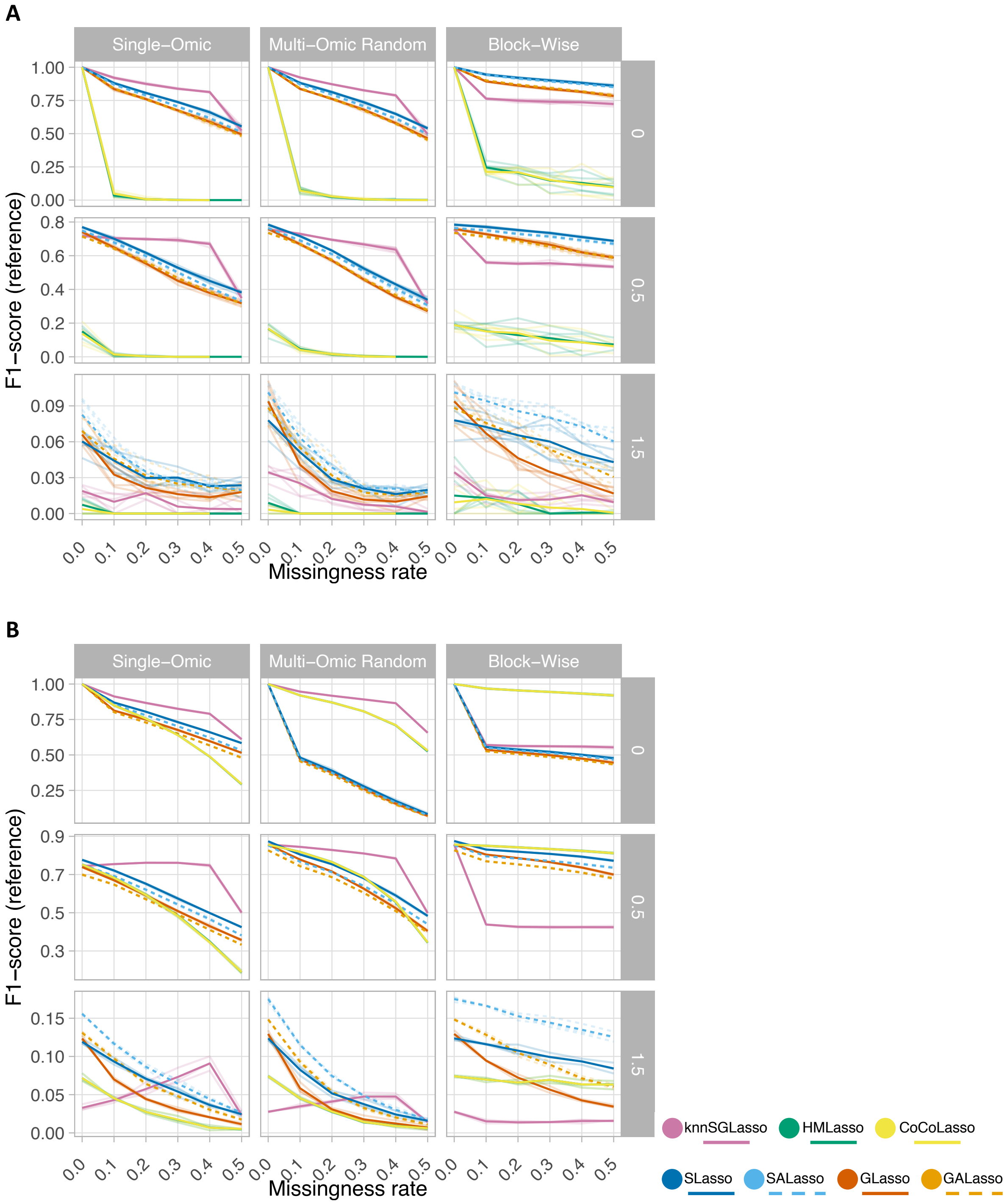

Supplementary Figure 6. Performance evaluation via F1-score. Network inference using full data was used as reference to calculate the F1-score per lasso model, **A)** TCGA-BRCA, **B)** TCGA-MIBC.

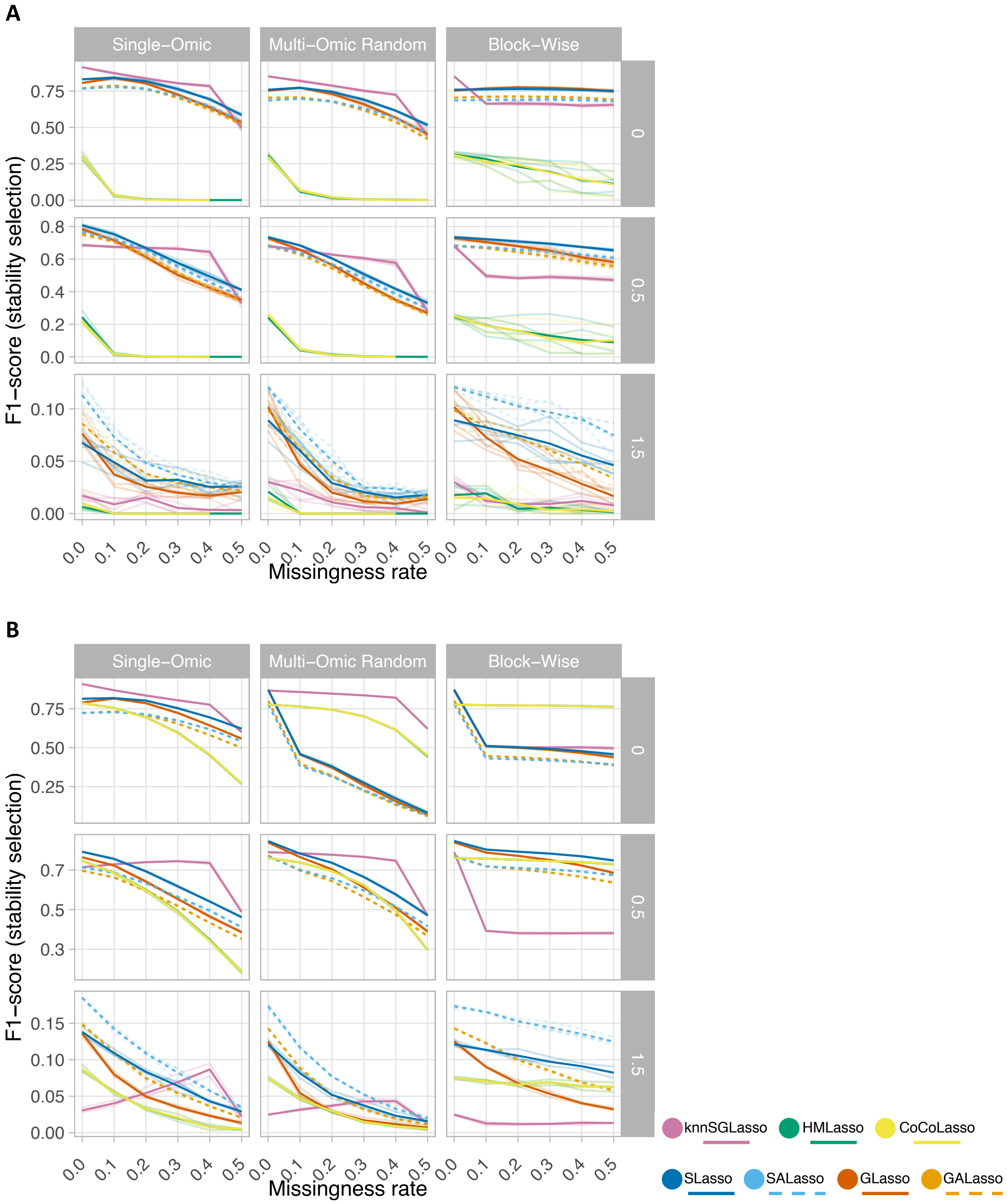

Supplementary Figure 7. Performance evaluation via F1-score. Network inference using KiMONo with stability selection (*k* = 100) over full data was used as reference to calculate F1-score for all lasso models, **A)** TCGA-BRCA, **B)** TCGA-MIBC.

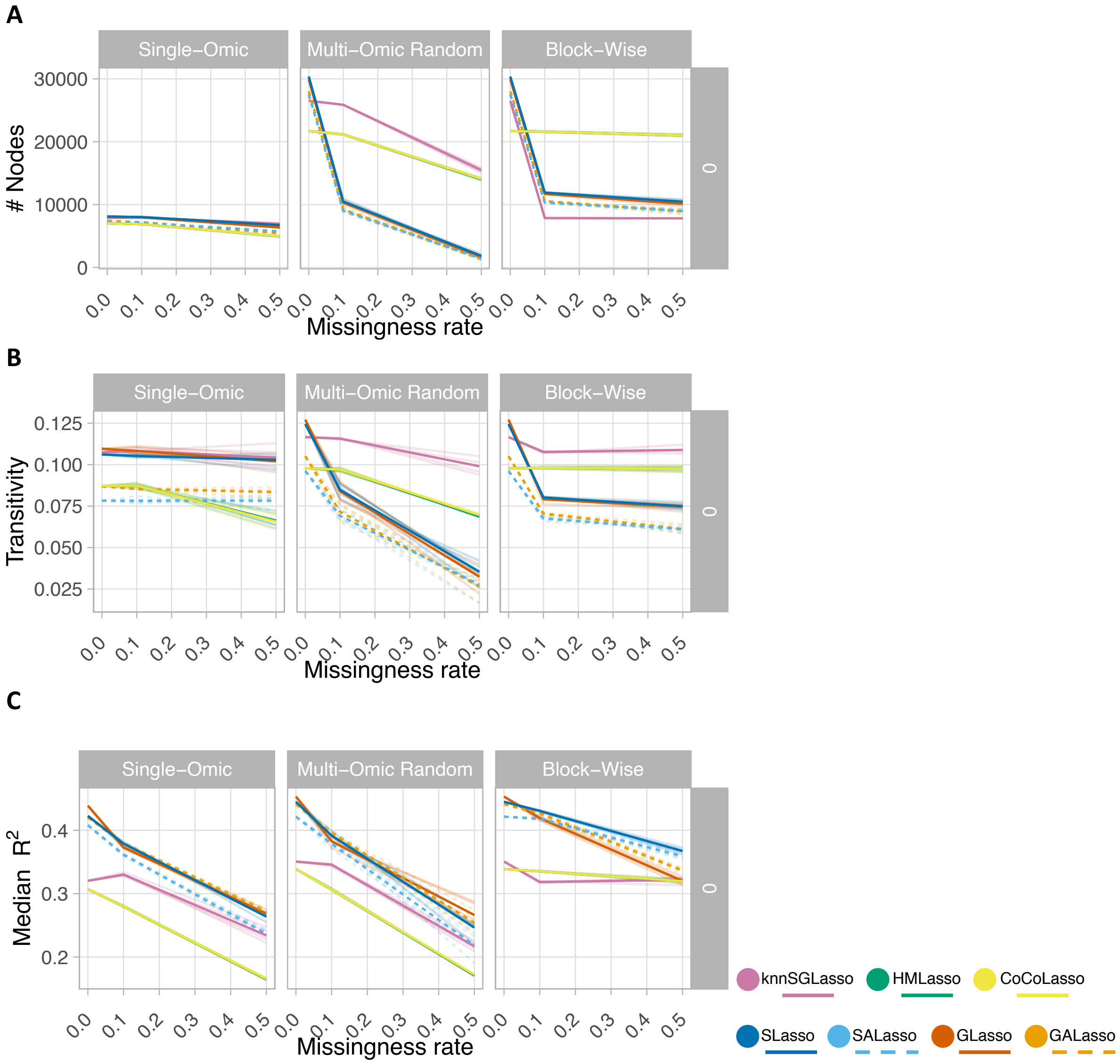

Supplementary Figure 8. Performance evaluation for TCGA-PRAD, **A)** Number of nodes indicating network size, **B)** Global clustering coefficient per network inferred, **C)** Median R^2 encompassing the model performance per network inferred.

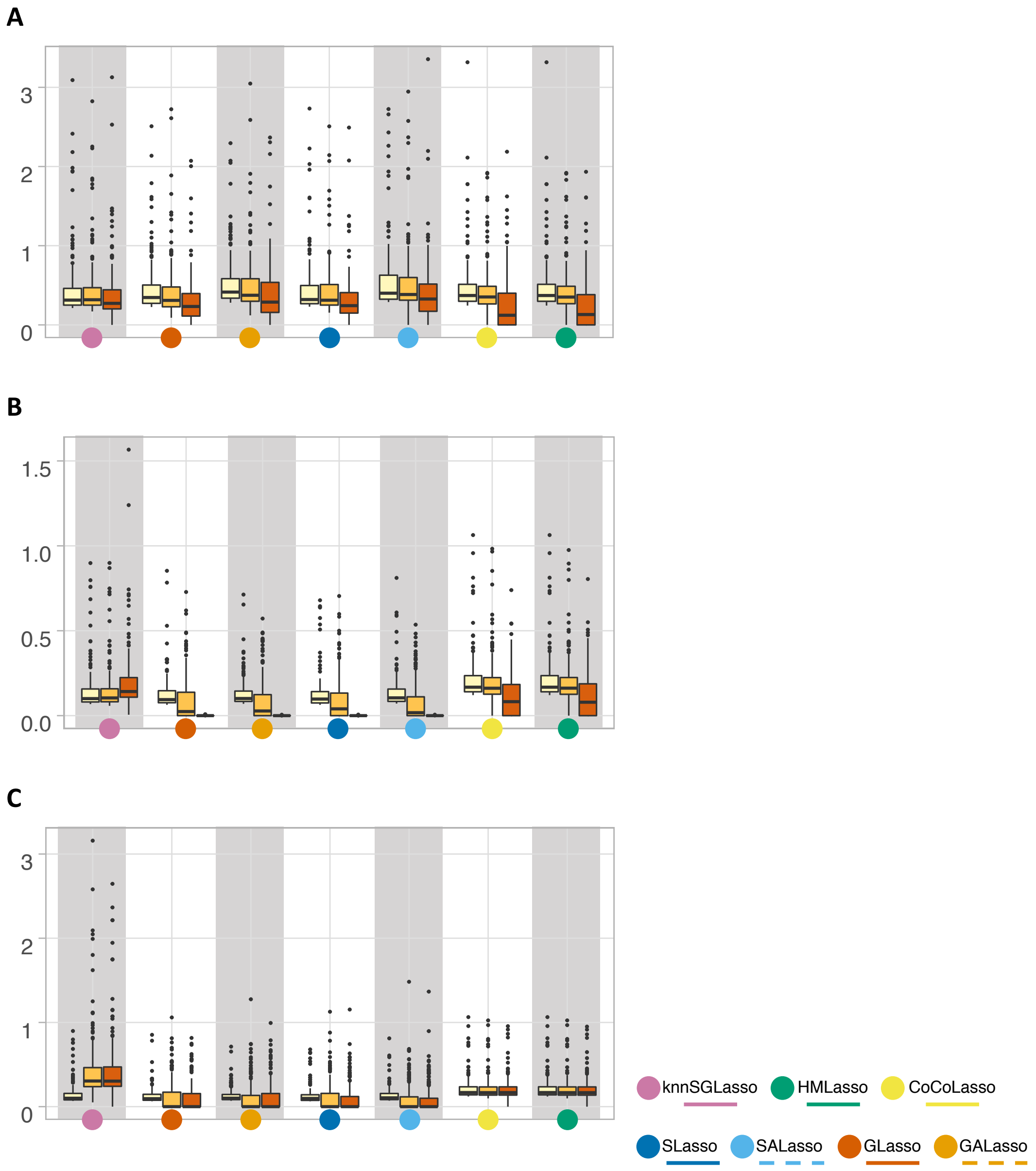

Supplementary Figure 9. Performance evaluation via betweenness centrality for TCGA-PRAD, **A)** Single-Omics experiment, **B)** Multi-omics experiment, **C)** Block-missingness experiment. All experiments were taken for 0%, 10%, and 50% missing ratios.

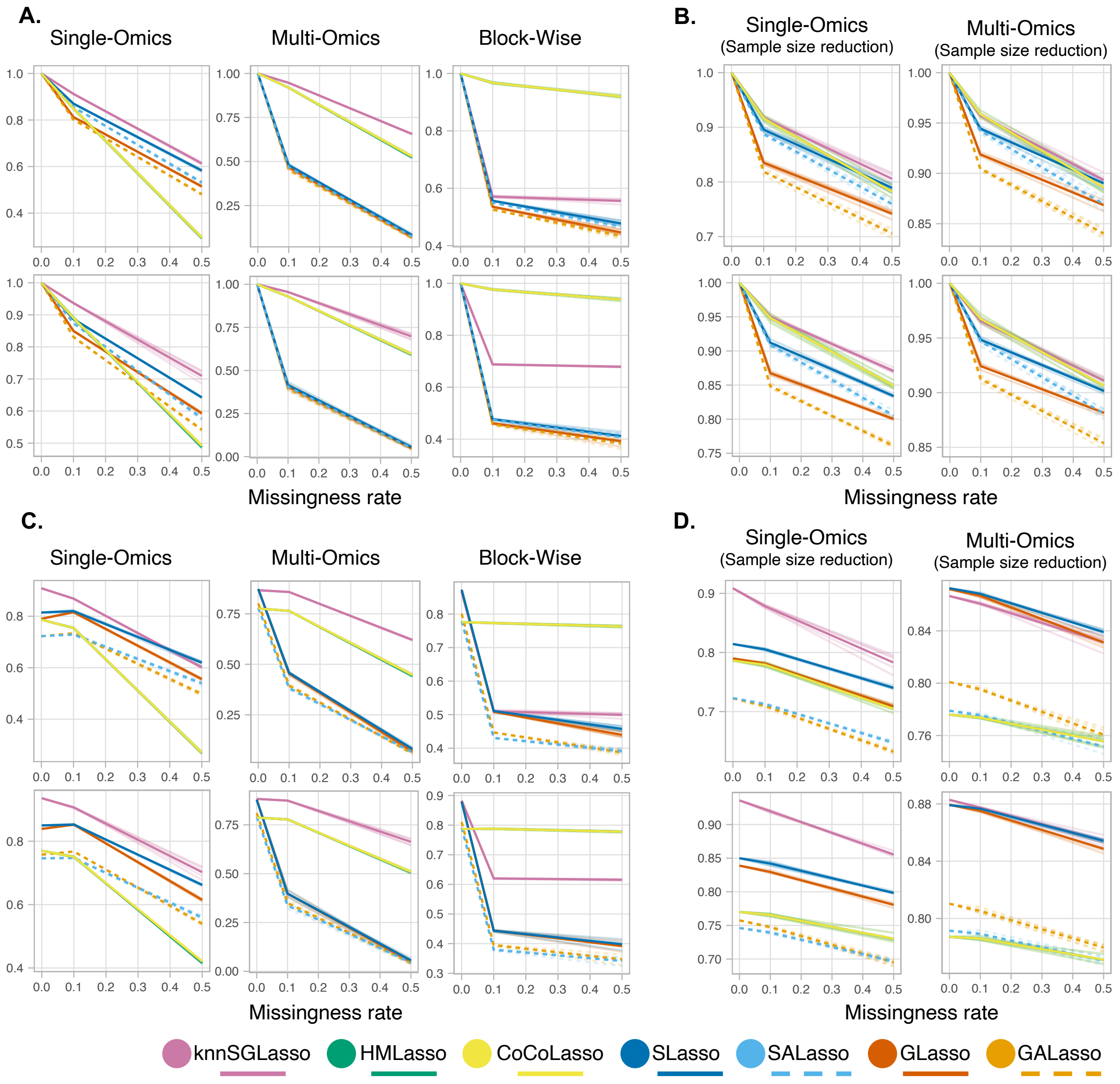

Supplementary Figure 10. Performance evaluation via F1-score for 10% and 50% missingness rate, **A)** & **B)** Experimental set-ups for TCGA-MIBC (top) and TCGA-PRAD (bottom) using full data as reference, **C)** & **D)** Experimental set-ups for TCGA-MIBC (top) and TCGA-PRAD (bottom) using stable networks.

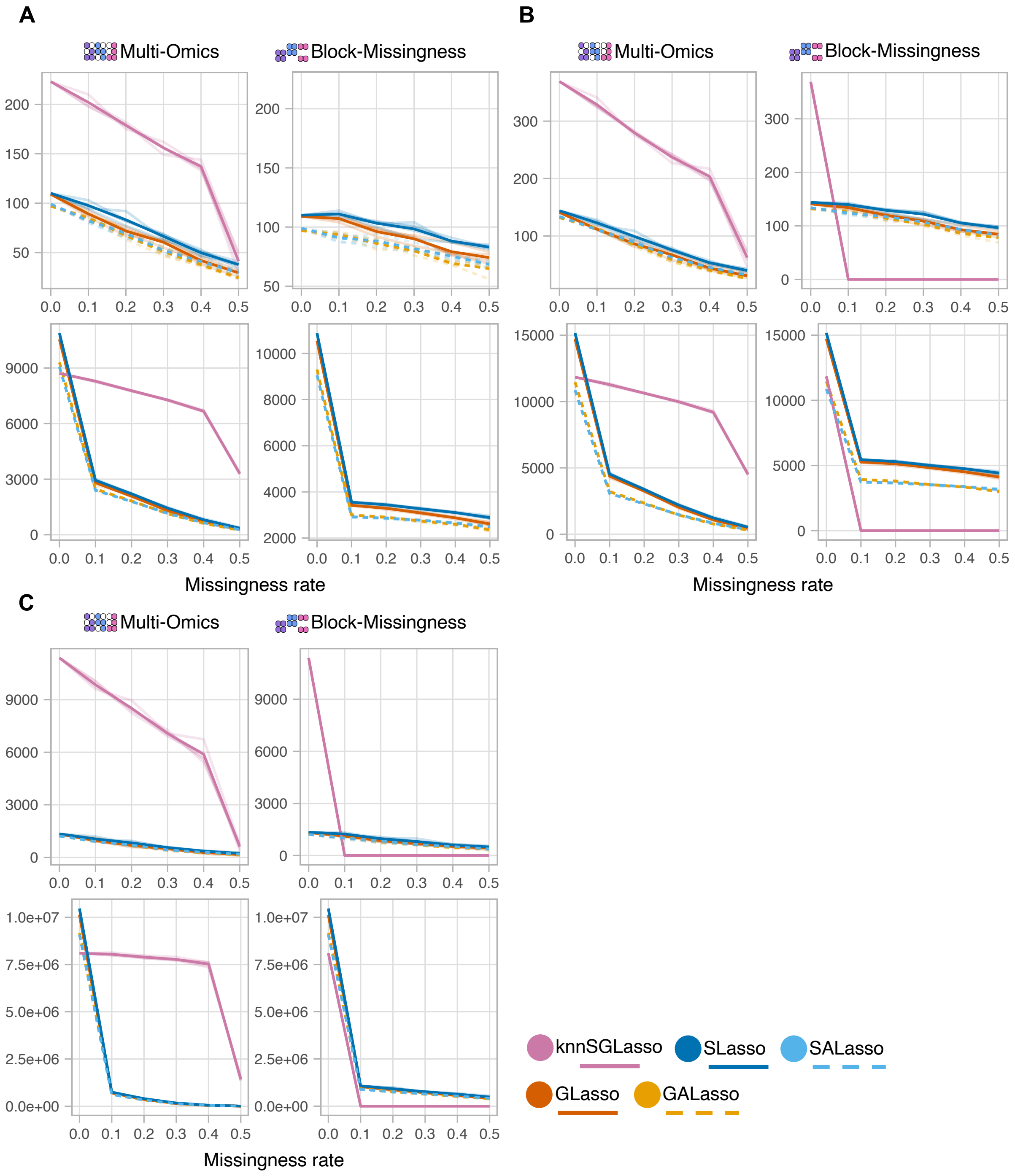

Supplementary Figure 11. Intra-layer evaluation via eQTM detection. **TCGA-BRCA** (top) and **TCGA-MIBC** (bottom). **A)** eQTM-linked genes detected by different inferred networks using different sparse. **B)** Number of total eQTMs detected by different inferred networks using different sparse models. **C)** Number of total eQTMs detected by Matrix eQTL based on the gene-methylation links obtained from the different inferred networks.
